# Glucosamine affects intracellular signalling through inhibition of mitogen-activated protein kinase phosphorylation in human chondrocytes

**DOI:** 10.1186/ar2307

**Published:** 2007-10-09

**Authors:** Anna Scotto d'Abusco, Valentina Calamia, Claudia Cicione, Brunella Grigolo, Laura Politi, Roberto Scandurra

**Affiliations:** 1 Department of Biochemical Sciences, Sapienza University of Roma, P.le Aldo Moro 5, 00185 Roma, Italy; 2 Laboratory of Immunology and Genetics, Istituto di Ricerca Codivilla Putti, Istituti Ortopedici Rizzoli, Via di Barbiano 1/10, 40136, Bologna Italy

## Abstract

The aim of this study was to determine the effects of glucosamine on matrix metalloprotease (MMP) production, on mitogen-activated protein kinase (MAPK) phosphorylation, and on activator protein (AP)-1 transcription factor activation in human chondrocytes. The human immortalized cell line lbpva55 and healthy human chondrocytes (obtained from healthy donors) were subjected to challenge with 10 ng/ml IL-1β after pretreatment with 2.5 or 10 mmol/l glucosamine. MMP mRNA expression levels were evaluated using quantitative real-time PCR, and MMP protein production levels were evaluated in the culture supernatant using ELISA. MAPK phosphorylation was evaluated using Western blotting. AP-1 transcription factor activation was evaluated by measuring AP-1 DNA-binding activity. After IL-1β stimulation, levels of MMP-1, MMP-3 and MMP-13 production were markedly increased. Treatment with 2.5 and 10 mmol/l glucosamine reduced expression of these metalloproteases. MMP expression is regulated by transcription factors such as the AP-1 complex, which is activated by phosphorylated MAPKs. IL-1β stimulated phosphorylation of c-jun amino-terminal kinase, p38 MAPK and extracellular signal-regulated kinase-1/2. Glucosamine inhibited c-jun amino-terminal kinase and p38 phosphorylation, and consequently c-jun binding activity. These findings demonstrate, for the first time, that glucosamine inhibits IL-1β-stimulated MMP production in human chondrocytes by affecting MAPK phosphorylation.

## Introduction

The pharmacological treatment of osteoarthritis (OA), a joint disorder characterized by slow, progressive degradation of the cartilage, includes analgesic agents and nonsteroidal antinflammatory drugs. During recent years there has been growing interest in alternative treatments for OA, such as glucosamine. In particular, glucosamine was found to be effective in reducing joint space narrowing compared with placebo in clinical trials conducted over a period of 3 years [1-4]. It was also found to be effective in decreasing pain compared with analgesic agents in OA of the knee [5,6]. A recent trial showed that glucosamine was ineffective in reducing pain in patients with severe knee OA, but it was more effective when it was used in combination with chondroitin sulphate in patients with moderate-to-severe pain [7].

Cartilage degradation in OA is due to an imbalance between synthesis and degradation of extracellular matrix components. Proinflammatory cytokines, such as IL-1β, which are produced in OA, trigger several biological effects by stimulating mitogen-activated protein kinase (MAPK) phosphorylation. The latter results in activation of transcription factors [8-10], which in turn upregulate the production of several molecules such as matrix metalloproteases (MMPs) and aggrecanases. Increased enzymatic activity of MMPs and aggrecanases is the major factor responsible for matrix degradation [11,12].

Several studies have examined the effects of glucosamine on MMP expression and activity in stimulated chondrocytes, obtained from various sources. The addition of glucosamine to cells appears to decrease the activity of MMPs [13-19]. Moreover, most *in vitro *studies conducted to elucidate the molecular basis of the effect of glucosamine on cartilage cells [20-24] demonstrated an anti-inflammatory and chondroprotective role for this molecule. However, the mechanisms responsible for these activities are not entirely understood.

To address whether glucosamine can inhibit production of MMPs by affecting IL-1β-induced MAPK activation, we investigated the phosphorylation of c-jun amino-terminal kinase (JNK), p38 and extracellular signal-regulated kinase (ERK)1/2 after pretreatment with glucosamine and stimulation with IL-1β. Moreover, we analyzed the activation of some activator protein (AP)-1 transcription factor components. We conducted the study both in the human immortalized chondrocyte cell line lbpva55 (derived from adult articular healthy cartilage), which has been demonstrated to be a useful tool for studying the biology of chondrocytes [25-27], and in human primary chondrocytes (HPCs) from healthy donors as a further control.

## Materials and methods

### Cell culture

lbpva55 cell culture was conducted as described previously [25]. Briefly, human immortalized chondrocytes, from the lbpva55 cell line, were grown to 80% confluence in Dulbecco's modified Eagle's medium (DMEM; Sigma, St. Louis, MO, USA) supplemented with L-glutamine, penicillin/streptomycin (HyClone, Logan, UT, USA) and gentamycin (Roche Diagnostic, Mannheim, Germany), along with 20% foetal bovine serum (FBS). The cells were then transferred in DMEM plus 10% FBS. After overnight incubation, the monolayer was rinsed with phosphate-buffered saline (PBS; Sigma) and incubated with culture medium containing 1% Nutridoma-SP (Roche). Medium was changed twice a week and the cells were split once. In these culture conditions, after 14 days the cells re-expressed the differentiated chondrocyte phenotype (namely collagen type IIA1 mRNA) [25].

HPCs were isolated from cartilage obtained from six healthy donors. Full informed consent was obtained from all donors and families.

Articular cartilages were aseptically dissected. Chondrocytes were obtained after sequential digestion with protease type IV (Sigma; 1 mg/ml) for 30 minutes and collagenase type II (Sigma; 1 mg/ml) for 90 minutes, both in Hank's medium (Hyclone). Chondrocytes were grown to 80% confluence in DMEM, supplemented as described above, along with 10% FBS. Experiments were performed with first passage cells in DMEM containing 1% FBS and were repeated in HPCs derived from the six donors, analyzing each sample separately.

### Cell treatment

lbpva55 cell line and HPCs were seeded in 60 mm plates at density of about 3 × 10^6 ^per plate. Cells were left untreated or treated with 10 ng/ml recombinant IL-1β (PeproTech House, London, UK) or pretreated for 2 hours with 2.5 or 10 mmol/l (0.54 and 2.16 mg/ml, respectively) glucosamine (Sigma) and then stimulated with 10 ng/ml IL-1β for 22 hours. Culture supernatants were collected and analyzed by ELISA, and cells were harvested and processed for quantitative real-time PCR.

To analyze early responsive proteins, JNK, p38, ERK1/2 and AP-1 components, lbpva55 cells and HPCs were pre-incubated for 2 hours in 2.5 or 10 mmol/l glucosamine containing medium and then stimulated with 10 ng/ml IL-1β for 15 minutes. Cells were harvested and conveniently processed for Western blot analysis or for DNA-binding activity.

### RNA extraction and reverse-transcription

Total RNA was extracted using TRIZOL reagent (Invitrogen, Carlsbad, CA, USA), in accordance with the manufacturer's instructions. Briefly, a confluent 60 mm plate, either of lbpva55 or HPCs, was washed with PBS and homogenized in 1 ml TRIZOL reagent. RNA was stored at -80°C until use.

cDNA was synthesized from 1 μg total RNA, using reverse transcriptase Improm II enzyme (Promega Corporation, Madison, WI, USA) in accordance with the manufacturer's instructions, and analyzed by quantitative real-time PCR.

### Real-time PCR

Quantitative real-time PCR analysis was performed using an ABI Prism 7300 (Applied Biosystems, Foster City, CA, USA). Amplification was carried out with 50 ng cDNA, in 96-well plates, using SYBR Green PCR Master mix (Applied Biosystems) in a 25 μl volume. Each sample was analyzed in triplicate. PCR conditions were as follows: 94°C for 10 minutes followed by 40 cycles of 94°C for 15 seconds and 60°C for 1 minute. Primers were designed using Primer Express software (Applied Biosystems) and were synthesized by Primm (Milan, Italy). The primer sequences are summarized in Table 1. The results were analyzed using Sequence Detection Systems software (Applied Biosystems), which automatically records the threshold cycle (C_t_). The untreated cell sample (control) was used as a calibrator; the fold change for control was 1.0. Target gene C_t _values were normalized against *GAPDH*. Data were analyzed using the 2^-ΔΔCt ^method and expressed as fold change compared to control.

**Table 1:** Sequences of primers used to quantify gene expression by real-time PCR

Gene	Primers	GenBank
GAPDH	Forward: GGAGTCAACGGATTTGGTCGTA	NM_002046
	Reverse: GGCAACAATATCCACTTTACCAGAGT	
MMP-1	Forward: GATGGACCTGGAGGAAATCTTG	NM_002421
	Reverse: TGAGCATCCCCTCCAATACC	
MMP-2	Forward: GCACCCATTTACACCTACACCAA	NM_004530
	Reverse: AGAGCTCCTGAATGCCCTTGA	
MMP-3	Forward: CCTGGTACCCACGGAACCT	NM_002422
	Reverse: AGGACAAAGCAGGATCACAGTTG	
MMP-8	Forward: GACCAACACCTCCGCAAATT	NM_002424
	Reverse: CCCCAAAGAATGGCCAAAT	
MMP-9	Forward: GGACGATGCCTGCAACGT	NM_004994
	Reverse: ACAAATACAGCTGGTTCCCAATC	
MMP-13	Forward: TTCTTGTTGCTGCGCATGA	NM_002427
	Reverse: TGCTCCAGGGTCCTTGGA	

GAPDH, glyceraldehyde-3-phosphate dehydrogenase; MMP, matrix metalloprotease.

### ELISA

For quantification of MMP levels in the culture medium, cells were treated as described above. Twenty-four hours after treatment, supernatants were collected and stored at -80°C until analysis using ELISA. Human MMP-1 ELISA kits were purchased from Chemicon International, Inc. (Temecula, CA, USA), and human MMP-3 and MMP-13 ELISA kits were purchased from Amersham Biosciences (GE Healthcare Europe, Milan, Italy). The experiments were performed in accordance with the manufacturers' instructions.

### Western blotting

To analyze MAPK phosphorylation, we performed Western blotting experiments. Cells, treated as described above, were washed with PBS and then scraped in 2× denaturing SDS buffer (Sigma). Extracts were heated to 100°C for 5 minutes and resolved on 10% SDS-PAGE. Gels were transferred to Hybond C membranes (GE Healthcare) by electroblotting (Bio-Rad Laboratories, Hercules, CA, USA) and probed with specific antibodies, in accordance with the manufacturers' instructions. Antibodies to JNK, phosphorylated-JNK and p38 were purchased from Santa Cruz Biotechnology, Inc. (Santa Cruz, CA, USA), antibodies to ERK1/2 and phosphorylated-ERK1/2 were from Biosource International (Camarillo, CA, USA), and antibodies to phosphorylated-p38 were from Chemicon International, Inc.

### AP-1 binding assay

Nuclear proteins were obtained from HPCs, treated as described above, using the Nuclear Extracts Kit (Active Motif, Carlsbad, CA, USA), in accordance with the manufacturer's instructions. Pellets were resuspended in 22 μl of Active Motif lysis buffer and proteins were measured (Bio-Rad Protein Assay). AP-1 consensus nucleotide binding activity from nuclear extracts (8 μg) was assessed using the TransAM AP-1 family kit (Active Motif), as recommended by the manufacturer. Nuclear extract was added to the immobilized oligonucleotides, followed by primary transcription factor antibody, secondary horse radish peroxidase (HRP)-conjugated antibody and HRP substrate, and colorimetric values (measured at 450 nm) were plotted.

### Statistical analysis

Each experiment was repeated at least three times. The statistical significance of the differences between mean values was determined using a two-tailed *t*-test. *P *≤ 0.05 was considered statistically significant. Where appropriate, results are expressed as the mean ± standard error.

## Results

### Effect of glucosamine on expression of MMPs in the lbpva55 cell line

**Figure 1:**
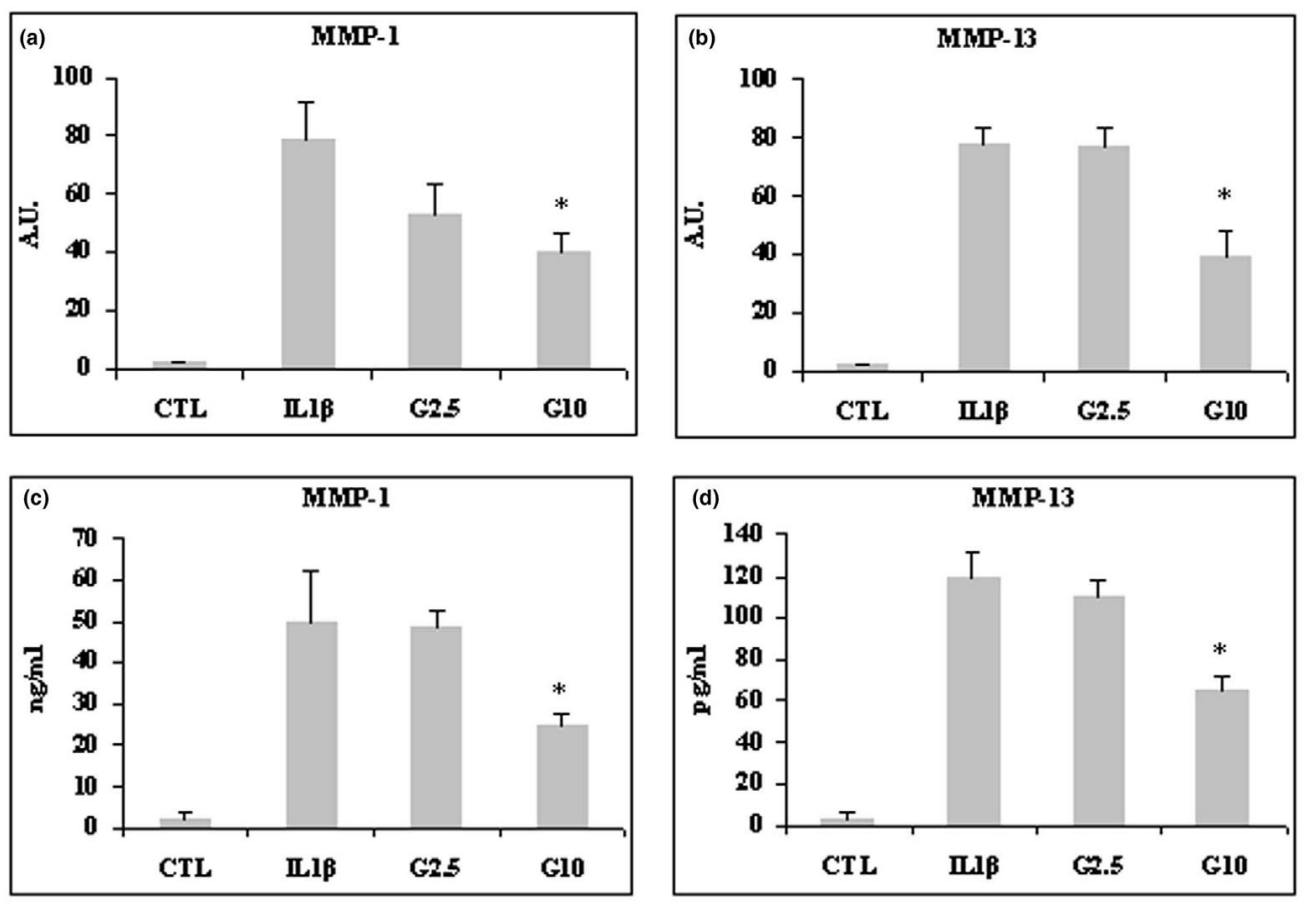
Effect of glucosamine on MMP-1 and MMP-13 expression in lbpva55 cells stimulated with 10 ng/ml IL-1β. Cells were pretreated for 2 hours with 2.5 or 10 mmol/l glucosamine (G2.5 and G10, respectively), and then stimulated with IL-1β for 22 hours. mRNA was extracted and analyzed by quantitative real-time PCR, and cell supernatant was analyzed by ELISA. Shown are **(a) **matrix metalloprotease (MMP)-1 and **(b) **MMP-13 mRNA levels, and **(c) **MMP-1 and **(d) **MMP-13 protein amounts. Quantitative real-time PCR results are expressed in relative arbitrary units (AU), and ELISA results are expressed in ng/ml or pg/ml. Results are expressed as mean ± standard error, obtained in three different experiments. *P ≤ 0.05. CTL, control.

After stimulation with IL-1β, MMP-1 and MMP-13 mRNA levels were markedly upregulated (both MMPs almost 80-fold). Pretreatment with 2.5 mmol/l and 10 mmol/l glucosamine inhibited MMP-1 and MMP-13 mRNA expression (Figure 1a,b), but only the treatment with 10 mmol/l glucosamine yielded a statistically significant effect (*P *< 0.05 for MMP1 and *P *< 0.03 for MMP-13). MMP-8 mRNA expression was not upregulated by IL-1β (data not shown). Consistent with quantitative real-time PCR findings, the ELISA assay demonstrated that levels of MMP-1 and MMP-13 protein secreted into the media were significantly decreased by 10 mmol/l glucosamine (*P *< 0.05; Figure 1c,d).

**Figure 2:**
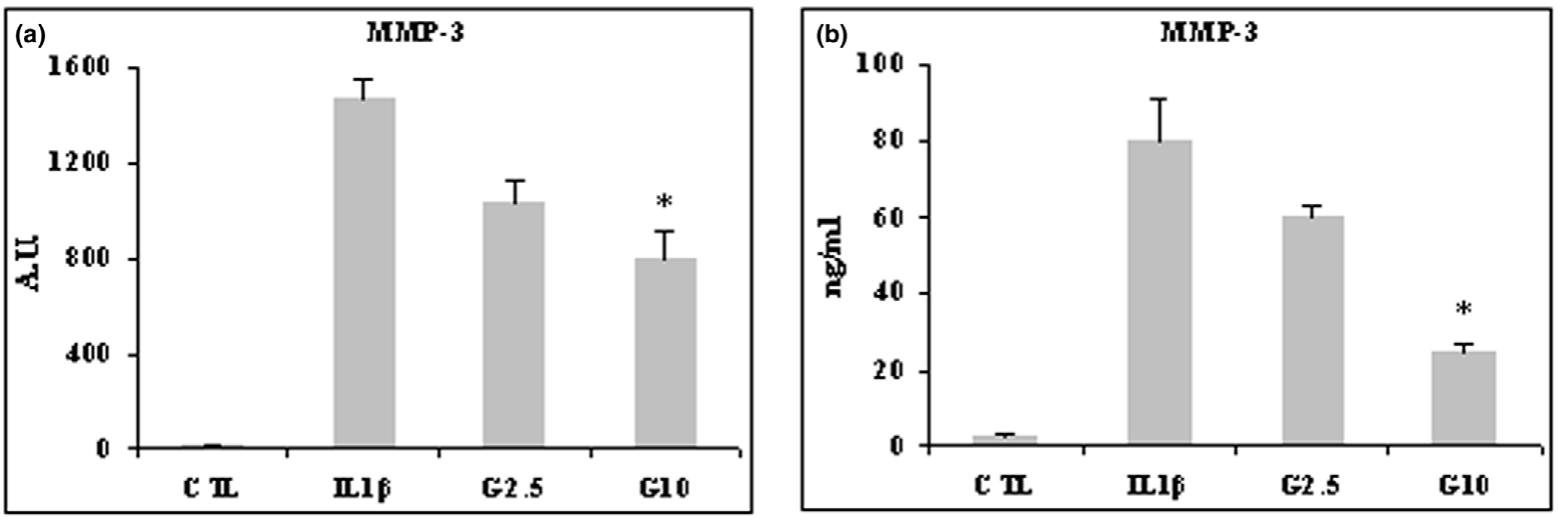
Effect of glucosamine on MMP-3 expression in lbpva55 cell line stimulated with 10 ng/ml IL-1β. Cells were pretreated for 2 hours with 2.5 or 10 mmol/l glucosamine (G2.5 and G10, respectively), and then stimulated with IL-1β for 22 hours. mRNA was extracted and analyzed by quantitative real-time PCR, and cell supernatant was analyzed by ELISA. Shown are **(a) **matrix metalloprotease (MMP)-3 mRNA and **(b) **MMP-3 protein levels. Quantitative real-time PCR results are expressed in relative arbitrary units (AU) and ELISA results are expressed in ng/ml. Results are expressed as mean ± standard error, obtained in three different experiments. *P ≤ 0.05. CTL, control.

IL-1β stimulated by 1,500-fold the expression of MMP-3 mRNA. This stimulation was significantly counteracted by 10 mmol/l glucosamine (*P *< 0.05; Figure 2a). IL-1β also stimulated secretion of MMP-3, which was counteracted by 2.5 mmol/l glucosamine and significantly so by 10 mmol/l glucosamine (*P *< 0.03; Figure 2b).

Levels of MMP-2 and MMP-9 mRNA expression were not upregulated by cytokine stimulation (data not shown) and their protein levels were not analyzed.

### Effect of glucosamine on expression of MMPs in HPCs

**Figure 3:**
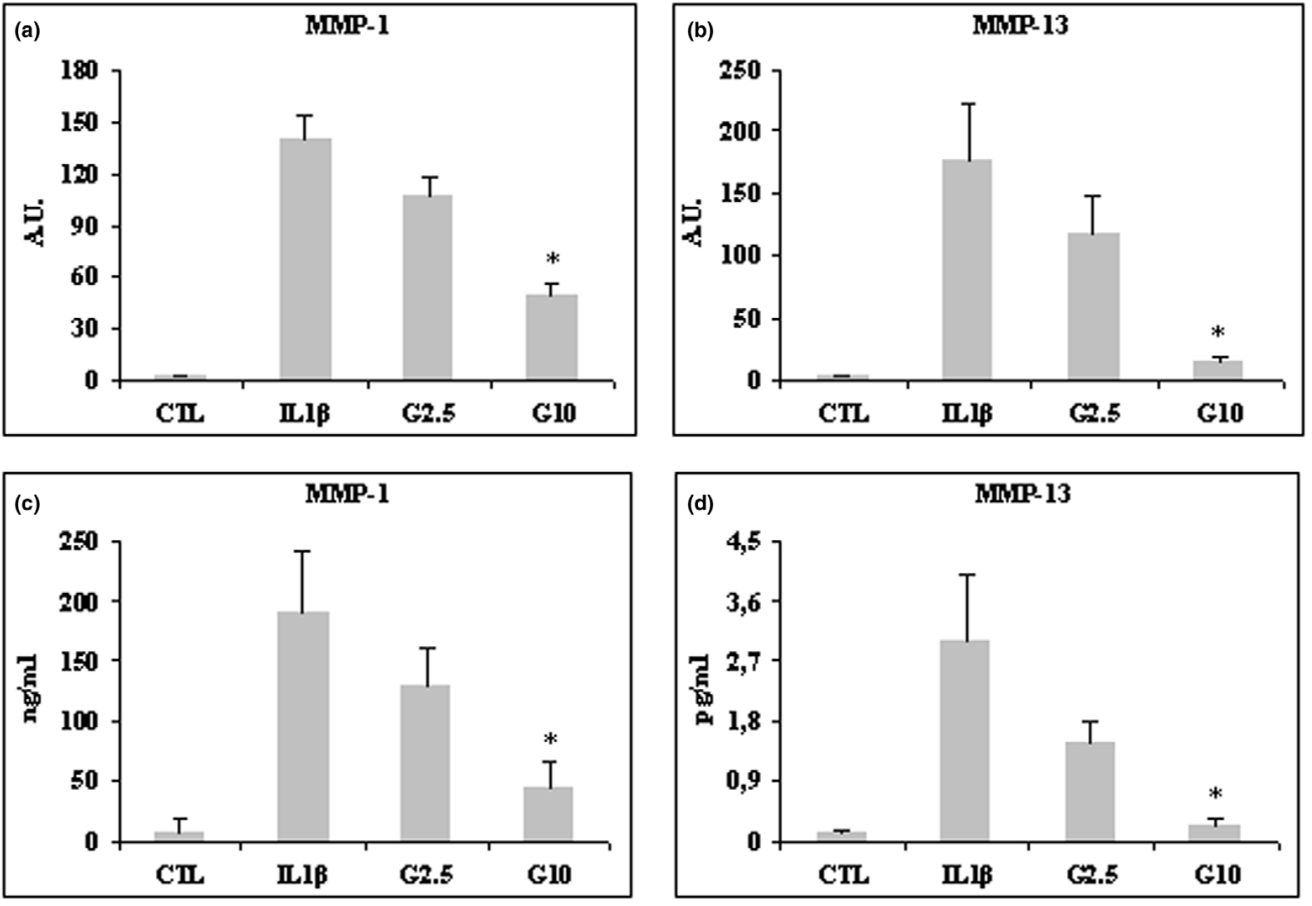
Effect of glucosamine on MMP-1 and MMP-13 expression in HPCs stimulated with 10 ng/ml IL-1β. Cells were pretreated for 2 hours with 2.5 and 10 mmol/l glucosamine (G2.5 and G10, respectively), and then stimulated with IL-1β for 22 hours. mRNA was extracted and analyzed by quantitative real-time PCR, and cell supernatant was analyzed by ELISA. Shown are **(a) **matrix metalloprotease (MMP)-1 and **(b) **MMP-13 mRNA levels, and **(c) **MMP-1 and **(d) **MMP-13 protein amounts. Quantitative real-time PCR results are expressed in relative arbitrary units (AU) and ELISA results are expressed in ng/ml or pg/ml. Results are expressed as mean ± standard error, obtained in six different experiments. *P ≤ 0.05. CTL, control; HPC, human primary chondrocyte.

**Figure 4:**
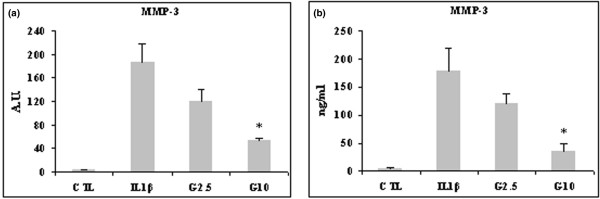
Effect of glucosamine on MMP-3 expression in HCPs stimulated with 10 ng/ml IL-1β. Cells were pretreated for 2 hours with 2.5 or 10 mmol/l glucosamine (G2.5 and G10, respectively), and then stimulated with IL-1β for 22 hours. mRNA was extracted and analyzed by quantitative real-time PCR, and cell supernatant was analyzed by ELISA. Shown are **(a) **matrix metalloprotease (MMP)-3 mRNA level and **(b) **MMP-3 protein level. Quantitative real-time PCR results are expressed in relative arbitrary units (AU) and ELISA results are expressed in ng/ml. Results are expressed as mean ± standard error, obtained in six different experiments. *P ≤ 0.05. CTL, control; HPC, human primary chondrocyte.

MMP-1, MMP-3 and MMP-13 were also stimulated by IL-1β in HPCs at both mRNA and protein levels, and the stimulation was counteracted by glucosamine treatment. MMP-1 mRNA expression level was stimulated 140-fold, MMP-3 180-fold and MMP-13 170-fold. All three MMPs were downregulated by 2.5 mmol/l and significantly so by 10 mmol/l glucosamine (*P *< 0.01 for MMP-1 and *P *< 0.05 for MMP-13 [Figure 3a,b] and *P *< 0.03 for MMP-3 [Figure 4a]). Levels of MMP-1 and MMP-3 secretion induced by IL-1β were higher compared than those of MMP-13. At any rate, 10 mmol/l glucosamine was effective in significantly downregulating all three MMPs (*P *< 0.05 for MMP-1 and *P *< 0.01 for MMP-13 [Figure 3c,d] and *P *< 0.03 for MMP-3 [Figure 4b]).

### Effect of glucosamine on IL-1β-induced phosphorylation of JNK, p38 and ERK1/2 MAP kinases in lbpva55 cell line and HPCs

**Figure 5:**
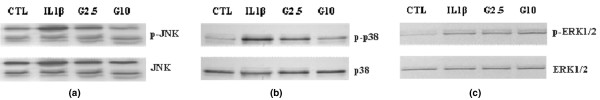
Effect of glucosamine MAPK phosphorylation in lbpva55 cells stimulated with 10 ng/ml IL-1β. Cells were pretreated for 2 hours with 2.5 or 10 mmol/l glocosamine (G2.5 and G10, respectively), and then stimulated with IL-1β for 15 minutes. Whole cell extract was prepared as described in Materials and methods. Proteins were resolved on SDS-PAGE, electrotransferred, and immunoblotted. Antibodies to **(a) **phosphorylated (p)-c-jun amino-terminal kinase (JNK) and total JNK, **(b) **p-p38 and total p38, and **(c) **p-extracellular signal-regulated kinase (ERK)1/2 and total ERK1/2 were used to visualize mitogen-activated protein kinase (MAPK) phosphorylation. Representative data from three independent experiments are shown. CTL, control.

**Figure 6:**
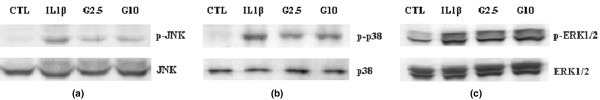
Effect of glucosamine MAPK phosphorylation in HPCs stimulated with 10 ng/ml IL-1β. Cells were pretreated for 2 hours with 2.5 or 10 mmol/l glucosamine (G2.5 and G10, respectively), and then stimulated with IL-1β for 15 minutes. Whole cell extract was prepared as described in Materials and methods. Proteins were resolved on SDS-PAGE, electrotransferred and immunoblotted. Antibodies to **(a) **phosphorylated (p)-c-jun amino-terminal kinase (JNK) and total JNK, **(b) **p-p38 and total p38, and **(c) **p-extracellular signal-regulated kinase (ERK)1/2 and total ERK1/2 were used to visualize mitogen-activated protein kinase (MAPK) phosphorylation. Representative data from six independent experiments are shown. CTL, control; HPC, human primary chondrocyte.

We analyzed the phosphorylation levels of three MAPKs, namely JNK, p38 and ERK1/2, by Western blotting. Time course experiments showed that 15 minutes of stimulation with IL-1β was able to induce phosphorylation of all three kinases analyzed in lbpva55 cells and HPCs (data not shown). Two hours of pretreatment with 2.5 or 10 mmol/l glucosamine prevented the phosphorylations of JNK (Figure 5a) and p38 (Figure 5b) in lbpva55 cells. Glucosamine was ineffective in counteracting the phosphorylation of ERK1/2 (Figure 5c). Similar findings were obtained in HPCs; IL-1β-induced phosphorylation of JNK and p38 was prevented by both 2.5 and 10 mmol/l glucosamine, which had no effect on ERK1/2 phosphorylation (Figure 6a,b,c).

### Effect of glucosamine on AP-1 transcription factor activation

**Figure 7:**
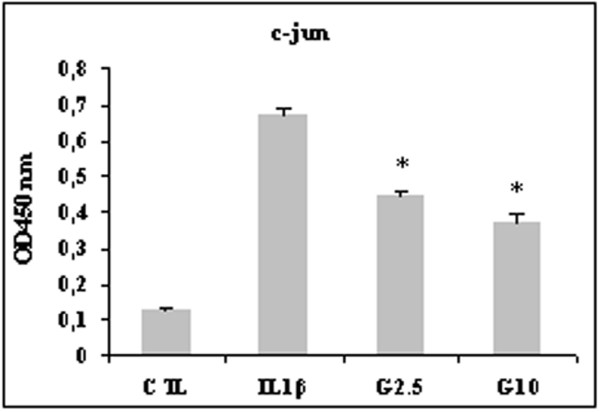
Effect of glucosamine on c-jun DNA-binding activity in HPCs stimulated with 10 ng/ml IL-1β. Cells were pretreated with 2.5 and 10 mmol/l glucosamine (G2.5 and G10, respectively) for 2 hours and then stimulated with IL-1β for 15 minutes. Nuclear extract was prepared as described in Materials and methods. Results are expressed as optical density (OD) measured at 450 nm and represent the mean ± standard error of data obtained in six different experiments. *P ≤ 0.05. CTL, control; HPC, human primary chondrocyte.

We next examined the effects of glucosamine on AP-1 components in HPCs. In our experimental conditions, 15 minutes of stimulation with IL-1β induced c-jun and junD DNA binding activity. Two hours of pretreatment with 2.5 and 10 mmol/l glucosamine significantly reduced c-jun DNA binding activity (*P *< 0.03; Figure 7), whereas junD binding activity was reduced to a lower degree (data not shown).

## Discussion

Imbalance between catabolic and anabolic factors in OA leads to degradation of articular cartilage. Catabolic factors include proinflammatory cytokines IL-1β and tumour necrosis factor-α, which stimulate intracellular signalling such as MAPK activation, which results in overproduction of several molecules, including MMPs [8-10]. In our experimental model, stimulation of the lbpva55 cell line with 10 ng/ml IL-1β resulted in upregulation of mRNA and protein expression of collagenases MMP-1 and MMP-13 and the stromelysin MMP-3. Pretreatment with 2.5 and 10 mmol/l glucosamine was able, to differing degrees, to downregulate mRNA and protein levels of MMP-1, MMP-3 and MMP-13.

These findings were confirmed in HPCs. In these cells as well, over-expression of MMP-1, MMP-3 and MMP-13 induced by IL-1β stimulation was downregulated by 2.5 and 10 mmol/l glucosamine pretreatment at both mRNA and protein levels. These findings are in agreement with those obtained by Nakamura and coworkers [18] in healthy and OA human chondrocytes. The lbpva55 cell line produces higher basal amounts of MMPs [27] as compared with those produced by HPCs, but MMP production is similarly stimulated by IL-1β and inhibited by glucosamine both in lbpva55 and HPCs.

To address the hypothesis that glucosamine can downregulate MMP production by affecting MAPK activation and therefore activation of transcription factors, we analyzed phosphorylation of JNK, p38 and ERK1/2 MAPKs. Phosphorylation of ERK1/2 was increased by IL-1β but was not decreased by glucosamine pretreatment. During the early phases of OA, proinflammatory cytokines promote chondrocyte proliferation. The ERK1/2 pathway is essential in processes that involve cellular proliferation and differentiation [28,29]. Chondrocytes stimulated *in vitro *with IL-1β mimic early phase OA, thus explaining the increased level of phosphorylation of ERK1/2. Nevertheless, glucosamine was ineffective in preventing phosphorylation of this kinase. JNK and p38 are involved in cellular stress; they are phosphorylated in response to proinflammatory cytokines, resulting in activation of transcription factors such as AP-1 complex [30], which are involved in expression of MMPs [8-10]. We found increased phosphorylation of JNK and p38 in samples stimulated with IL-1β, and decreased phosphorylation level in samples pretreated with 2.5 or 10 mmol/l glucosamine and then stimulated with IL-1β. Moreover, c-jun and junD activity were affected by glucosamine pretreatment in HPCs. Both c-jun [8-10] and junD [31] are involved in transcription of MMPs; inhibition of their activity is in accordance with decreased MMP production. Transcription of MMPs is also under the control of nuclear factor-κB [8]. Other investigators previously showed that glucosamine can interfere with nuclear factor-κB activation [22]. In our laboratory we confirmed that 2.5 and 10 mmol/l glucosamine prevent the migration into the nucleus of a nuclear factor-κB subunit, namely p65 (data not shown).

The decreased phosphorylation MAPKs may be explained by the role played by glucosamine in O-glycosylation of some proteins. Glucosamine, when it enters into the cells, can undergo several fates, one of which is N-acetylation; the resulting N-acetylglucosamine can modify several proteins by coupling itself to the serine or threonine residues of those proteins. These include kinases, phosphatases, transcription factors and metabolic enzymes, among others. O-glycosylation is thought to act in manner analogous to phosphorylation; in fact, O-glycosylation levels respond to cellular signals [32,33]. O-glycosylation and phosphorylation have been shown to be reciprocal on some proteins [32]. In other proteins, glycoforms and phosphoforms are distinct, although the sites are a few amino acids from each other [32].

We can speculate that O-glycosylation on JNK and p38 MAPKs inhibits their phosphorylation by utilizing the same sites, either by steric hindrance or modulation of the local protein structure. O-glycosylation on the ERK1/2 kinase could utilize different sites or sites localized far away from those of phosphorylation; this could explain why glucosamine does not affect ERK1/2 phosphorylation.

Our findings on inhibition of MAPK phosphorylation by glucosamine are not in agreement with those reported by Shikhman and coworkers [20], who found that N-acetylglucosamine counteracted the stimulations exerted by IL-1β on human chondrocytes, but its action was not associated with decreased phosphorylation of MAPKs. This discrepancy may be explained by the time period considered. Shikhman and coworkers analyzed phosphorylation 24 hours after IL-1β stimulation, whereas we examined MAPK phosphorylation 15 minutes after IL-1β stimulation, which is the best time to identify JNK, p38 and ERK1/2 phosphorylation because it takes into account the fact that MAPK phosphorylation is an early event in cell stimulation.

We analyzed the mRNA expression of gelatinases MMP-2 and MMP-9, but lbpva55 cells were not stimulated by IL-1β to over-produce these molecules. This finding is in agreement with those reported by Chan and coworkers [34], who did not identify upregulation of MMP-2 and MMP-9 in bovine chondrocytes stimulated with IL-1β, and with those reported by Duerr [35] and Soder [36] and their colleagues, who obtained similar results in human chondrocytes. In contrast, equine chondrocytes stimulated with lipopolysaccharide exhibited upregulated MMP-2 and MMP-9 protein levels [17].

Our experiments were performed with 2.5 and 10 mmol/l glucosamine; 10 mmol/l was more effective than 2.5 mmol/l in terms of MMP expression. Nevertheless, 2.5 mmol/l was almost as 10 mmol/l glucosamine in terms of affect on MAPK phosphorylation and AP-1 activation. The concentrations we used of both glucosamine and IL-1β are rather high and supraphysiological. As such, the results obtained are not reflective of the *in vivo *situation that occurs following oral administration of glucosamine in OA. Neither are they representative of the biological response that may be achieved. However, it was not our intention to model directly the *in vivo *situation; rather, our purpose was to seek an explanation for the effects of glucosamine on the biology of chondrocytes *in vitro*. The biology of the cell *in vitro *is affected by various factors, including cell medium. Our cell medium contains 25 mmol/l glucose; glucosamine utilizes glucose transporters to be taken up by the cells, competing with glucose [37,38]. Therefore, to appreciate the effect of glucosamine in the presence of high glucose concentrations, it was necessary to use a high concentration of glucosamine. Several authors utilize 10 ng/ml IL-1β and 10 mmol/l glucosamine [20-22,24], but other authors utilize lower glucosamine concentrations and lower IL-1β concentrations or culture medium containing a low concentration of glucose [13,16,18,23].

## Conclusion

We showed that pretreatment of human chondrocytes with glucosamine inhibited IL-1β-induced expression of MMP-1, MMP-3 and MMP-13, which are classical markers of inflammation and cartilage degradation in OA joints. It achieved this by affecting phosphorylation of JNK and p38 MAPKs and consequently inhibiting c-jun and junD activation. Other compounds, too, have been shown to inhibit production of MMPs by affecting MAPK activation in chondrocytes [39,40], but no explanation has yet been reported. Further investigations are required, and are in progress in our laboratory, to establish the mechanism utilized by glucosamine to inhibit MAPK phosphorylation.

## Abbreviations

AP = activator protein; C_t _= threshold cycle; DMEM = Dulbecco's modified Eagle's medium; ERK = extracellular signal-regulated kinase; FBS = foetal bovine serum; HPC = human primary chondrocyte; IL = interleukin; JNK = c-jun amino-terminal kinase; MAPK = mitogen-activated protein kinase; MMP = matrix metalloprotease; OA = osteoarthritis; PBS = phosphate-buffered saline; PCR = polymerase chain reaction.

## Competing interests

The authors declare that they have no competing interests.

## Authors' contributions

ASdA conceived the design of the study, carried out the cell cultures, performed quantitative real-time PCR, coordinated and trained others to perform the experiments, participated in statistical analysis, and coordinated all phases of manuscript writing. VC carried out ELISA and Western blot experiments, and participated in cell cultures and in statistical analysis. CC carried out quantitative real-time PCR experiments, and participated in cell cultures and in statistical analysis. BG carried out cell line immortalization and helped to draft the manuscript. LP and RS coordinated the laboratory work, participated in data analysis and helped to draft the manuscript. All authors read and approved the final manuscript.
